# Effect of Different Hot-Pressing Pressure and Temperature on the Performance of Titanium Mesh-Based MEA for DMFC

**DOI:** 10.3390/membranes12040431

**Published:** 2022-04-16

**Authors:** Xingxing Wang, Yujie Zhang, Yu Zhu, Shuaishuai Lv, Hongjun Ni, Yelin Deng, Yinnan Yuan

**Affiliations:** 1 School of Mechanical Engineering, Nantong University, Nantong 226019, China; wangxx@ntu.edu.cn (X.W.); 2009310020@stmail.ntu.edu.cn (Y.Z.); lvshuaishuai@ntu.edu.cn (S.L.); 2 School of Rail Transportation, Soochow University, Suzhou 215131, China; yuanyn@suda.edu.cn

**Keywords:** DMFC, MEA, titanium mesh, hot-pressing, Ti mesh, metal mesh, membrane electrode assembly, direct methanol fuel cell

## Abstract

The hot-pressing process of the membrane electrode assembly (MEA) is one of the research hotspots in the field of the fuel cell. To obtain suitable titanium mesh-based MEA hot pressing process parameters, titanium mesh was used as electrode substrate material. The anode and cathode of MEA were prepared by the drip-coated method, and the titanium mesh-based MEA was prepared under different hot-pressing pressure and temperature, respectively. The performance of titanium mesh-based MEA was studied by morphological observation, elemental analysis, thickness measurement, single cell test and numerical fitting analysis. The results demonstrated that: with increasing hot-pressing pressure from 0 MPa to 10 MPa, the forming thickness of titanium mesh-based MEA is getting thin gradually, and the peak power density of titanium mesh-based MEA first increased and then gradually decreased; with increasing hot-pressing temperature from 115 °C to 155 °C, the peak power density of titanium mesh-based MEA enhanced at the beginning and then also gradually decreased. Under the premise of a hot-pressing time of 180 s and the optimal operating temperature of DMFC of 60 °C, the appropriate hot-pressing process conditions of titanium mesh-based MEA are a hot-pressing pressure of 5 MPa and a hot-pressing temperature of 135 °C. The results can provide a technological reference for the preparation of titanium mesh MEA for DMFC.

## 1. Introduction

Fuel cell (FC) is a high-efficiency power generation device that directly converts the chemical energy of fuel into electrical energy through an electrochemical reaction without combustion [1,2]. Compared with other energy conversion methods, FC has many advantages: (1) In theory, as long as fuel is continuously supplied, FC can continuously generate electricity; (2) FC is clean, pollution-free, low in noise, and has a modular structure. It is suitable for various power requirements; (3) it has high energy conversion efficiency because it is not limited by the Carnot cycle. The theoretical energy conversion efficiency of FC is as high as 90%, which is better than the conventional power generation, and other chemical power sources; (4) FC does not need to be connected to the grid for power generation and has robust distribution. It is suitable for power supply in remote and inconvenient areas; (5) FC has a small size, strong mobility, convenient maintenance, and a short production cycle [3,4,5]. The fuel of FC can use hydrocarbons such as H_2_, NH_3_, N_2_H_4_, alcohol, aldehyde, or its reformed gas rich in H_2_ after the reformation, and the oxidant can generally use pure O_2_ or air. FC is divided into phosphoric acid fuel cell (PAFC), alkaline fuel cell (AFC), solid oxide fuel cell (SOFC), molten carbonate fuel cell (MCFC), and proton exchange membrane fuel cell (PEMFC), according to the type of electrolyte or fuel. PEMFC derives an alcohol-reforming fuel cell (ARFC) and direct alcohol fuel cell (DAFC) according to the different fuels used. DAFC mainly includes direct methanol fuel cell (DMFC) and direct ethanol fuel cell (DEFC). DMFC has always been the focus of research and development [6,7].

DMFC has the advantages of high volumetric energy density, simple system structure, short start-up time, high operational reliability, and convenient fuel replenishment. DMFC can also supply power continuously for a long time; thus, it has become the most promising alternative power source for the widely used batteries [8]. DMFC is suitable as a mobile power supply and portable power supply and has broad application prospects in communication, transportation, and national defense. In the past few years, the development and application of DMFC technology have developed rapidly. Many universities, research institutes, and major companies have spent huge manpower and material resources on DMFC research and development. Most of the research is to continue the flat structure of PEMFC. The membrane electrode assembly (MEA) is placed between two metal meshes and sealed with elastic gaskets. The outermost bipolar plate is stainless steel or machined graphite plate with flow channels [9]. However, DMFC still faces methanol leakage, electrode poisoning, and lack of self-storage fuel [10].

MEA is the core component of DMFC. It is usually composed of an anode, electrolyte membrane, and cathode. The electrolyte membrane generally adopts a perfluoro sulfonic acid proton exchange membrane, such as the Nafion series membrane produced by DuPont in the United States. The electrode usually consists of a gas diffusion layer and a catalytic layer. The catalytic layer is where the electrochemical reaction occurs, and the commonly used catalysts for the anode and cathode are PtRu and Pt noble metal systems. The gas diffusion layer supports the catalytic layer, collecting current and transferring materials in the MEA. It is generally made of conductive porous materials, and most of them are carbon paper, carbon cloth, and metal mesh, whose surface is leveled by carbon powder [11].

**Figure 1:**
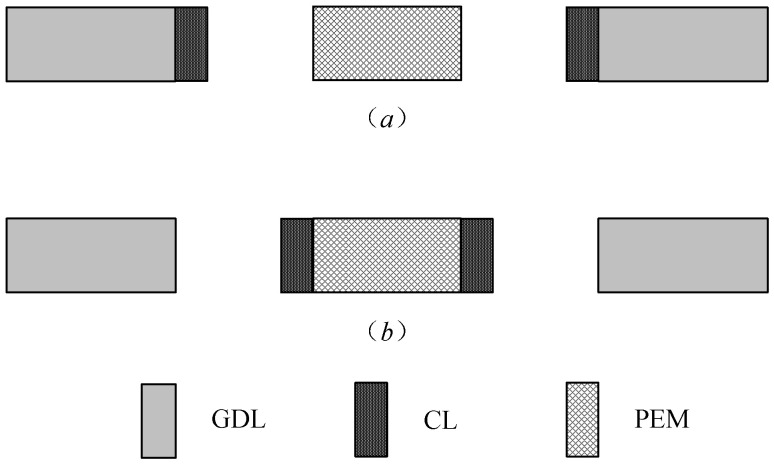
Schematic diagram of the preparation process of MEA. (**a**) GDE-PEM approach; (**b**) CCM-GDL approach.

The main preparation methods of MEA can be divided into (a) GDE-PEM and (b) CCM-GDL, two processes according to their preparation process. The summary drawing is shown in the Figure 1.

Type (a) is to prepare the catalytic layer (CL) on the gas diffusion layer (GDL) to form the gas diffusion electrode (GDE); GDE and proton exchange membrane (PEM) are then hot-pressed together to form MEA. The advantages of this approach are that it is easy to scale up and mass-produce, and the structure or size of both GDE and MEA can be changed very flexibly. Type (b) is to prepare the CL on the PEM and obtain the catalyst-coated membrane (CCM), then, laminate CCM and GDL together to form MEA. The advantage is that the structure of the catalytic layer and the gas diffusion layer can be optimized separately, and the catalyst or Nafion polymer will not permeate into the gas diffusion layer during the preparation process to cause mass transfer resistance [12].

Whether the MEA is prepared by the GDE-PEM route or the CCM-GDL route, the hot-pressing process is widely used when the MEA is finally formed. The hot-pressing method is widely used in the MEA forming process, and the parameters of the hot-pressing process are widely selected. However, some scholars have studied the hot-pressing process conditions for MEA with different PEM and GDL. The research of Zhang et al. [13] demonstrates that for the carbon paper-based MEA with the Nafion 117 membrane, the output power density of the MEA prepared with the hot-pressing pressure of 12.0 MPa, the hot-pressing time of 90 s, and the hot-pressing temperature of 135 °C is higher than that of the MEA prepared at the hot-pressing temperature of 125 °C. On the other hand, the output power density of the MEA prepared with the hot-pressing temperature of 135 °C, the hot-pressing time of 90 s, and the hot-pressing pressure of 8.0 MPa is higher than those of the MEA prepared at the hot-pressing pressure of 4.0 MPa, 12.0 MPa and 16.0 MPa; the output power density of the MEA prepared at 135 °C, 8.0 MPa, and 90 s is higher than that of the MEA prepared at 50 s and 180 s. Chen et al. [14] study the effects of two hot-pressing pressure conditions of 0.5 MPa and 10 MPa on the performance of MEA. Under the premise of a hot-pressing temperature of 120 °C and holding time of 2 min to 3 min, the Nafion 117 membrane carbon cloth-based MEA can obtain higher porosity and higher output power density when using 0.5 MPa hot-pressing pressure. Yildirim et al. [15] study the effect of hot-pressing pressure conditions on the performance of MEA. E-TEK commercial electrodes and Nafion 117 membranes are used to prepare MEA. The results demonstrate that the output power density of the MEA prepared with the hot-pressing pressure of 1.0 MPa is higher than those of the MEA prepared with the hot-pressing pressure of 0.2 MPa and 2.9 MPa. Jung et al. [16] study the effect of the heat treatment process conditions after hot-pressing on the performance of MEA. Nafion 117 membrane and carbon paper are used for MEA preparation. The results demonstrate that the MEA with the heat treatment temperature of 130 °C obtains the highest power density. The research of Liang et al. [17] demonstrates that when using Nafion 115 membrane and ELAT commercial electrode to prepare MEA, the hot-pressing pressure of 4 MPa, the hot-pressing temperature of 135 °C, and the hot-pressing time of 3 min, 30 min, and 60 min have slight effects on the power density performance of the battery; the effects of hot-pressing temperatures of 135 °C and 160 °C on the performance of MEA are also studied, and the results demonstrate that 135 °C is the best hot-pressing temperature. Oedegaard et al. [18] study the effect of different GDL materials on the performance of MEA. The MEA is prepared by hot-pressing with the Nafion 117 membrane. The type A carbon cloth from E-TEK company, type B carbon cloth from E-TEK company, type TGP-H-090 carbon paper from Toray company, and stainless-steel woven mesh from Filtertechnik GmbH Willy Spee company (107 mesh × 60 mesh) are selected, respectively. The results demonstrate that when GDL is stainless-steel woven mesh, the output power density of DMFC is the highest, reaching 15.8 mW·cm^−2^, which means that the best material for GDL is stainless-steel woven mesh. Stähler et al. [19] use the same hot-pressing process for the complete slot die-coated MEA(CC-MEA) on the polytetrafluoroethylene (PTFE) substrate and the Nafion hot-pressing MEA(HP-MEA), and find that while the hot-pressing temperature is 130 °C, the hot-pressing pressure is 5 MPa and the hot-pressing time is 2 min to 3 min; the absolute impedance of the complete slot die-coated MEA can converge to (0.034 ± 0.001) Ω·cm^2^, which can improve the current density more than HP-MEA. Shen et al. [20] demonstrate that optimizing the preparation temperature and pressure of the membrane electrode can effectively improve the performance of DMFC. Nafion 115 membrane and carbon paper are used for MEA preparation. MEA with hot-pressing pressure conditions of 80 kgf·cm^−2^ (approximately equal to 0.8 MPa) obtained a higher peak power density than 40 kgf·cm^−2^ (approximately equal to 0.4 MPa). With increasing hot-pressing temperature from 140 °C to 170 °C, the peak power density of carbon paper-based MEA increased successively. The research of Wang et al. [21] demonstrated that the hot-pressing temperature of 135 °C is more appropriate for making the titanium mesh MEA under hot-pressing under 5 MPa for 180 s. This is a brief introduction to the previous research work by the authors of this paper through a conference article.

Carbon paper is brittle, and carbon cloth is difficult to form, while the metal mesh is easy to form and can prepare MEA of different shapes. Yu et al. [22] roll self-sealing tubular MEA by misplacement and lap bonding of a cathode, plate electrolyte film, and anode, using rolling hot-pressing technology, carbon cloth coated with the catalyst as negative and anode, and Nafion membranes as electrolyte film. Ishida et al. [23] prepare microtubular DMFC with the Flemion tubular membranes produced by Asahi. The inner diameter of the membrane is 0.3 mm, and the outer diameter is 0.6 mm. Pt is deposited on the outside of the tubular membrane by electroless plating. Qiao et al. [24] use Flemion’s tubular film produced by Asahi and perform two steps of electroless plating and electroplating to prepare microtubular DMFC. Yazici [25] proposes the concept of a “replaceable cartridge fuel cell.” A permeable tubular structure or a rod with machined grooves on the surface is used as the battery anode, and the outer surface is coated with Pt catalyst. Then the tubular Nafion membrane is covered, and the cathode catalyst and cathode gas diffusion layer are coated and it is placed into a heat-shrinkable porous tube and heated to seal. A prototype tubular fuel cell with a diameter of 4 mm and a length of 5 cm is developed. Shao et al. [26] use a titanium mesh tube with a diameter of 3 mm provided by Heggemann to prepare a tubular cathode. The titanium mesh tube, the cathode gas diffusion layer, and the cathode catalyst layer are sequentially formed from the inside to the outside. In addition, the titanium mesh is used to make PtRuO_x_/Ti anode. Ni et al. [27] use a tubular metal titanium mesh as a support and successively prepare a gas diffusion layer, a Pt/C catalyst layer, and Nafion membranes on its outer surface by a dip-coating process. A tubular cathode is prepared, and the constant voltage discharge test results after about 100 h of working time demonstrate that the tubular titanium-based cathode has excellent electrochemical stability. Compared with the flat DMFC, the tubular DMFC has the advantages of fuel storage and higher volume power density. However, it also has the disadvantages of a complicated manufacturing process, complex molding, and the failure of large-scale commercial application of the tubular electrolyte membrane.

There is no detailed report on the research of the hot-pressing process conditions of titanium mesh-based MEA, beside the authors’ own previous research [20]. At the same time, titanium mesh-based MEA can be used to prepare special-shaped DMFC with different shapes, such as tubes. To determine the appropriate hot-pressing process conditions of titanium mesh-based MEA, combining the research status of hot-pressing process parameters of carbon paper-based MEA and carbon cloth-based MEA, the plate-shaped titanium mesh-based MEA is used as this paper research object. The effect of different hot-pressing pressure and temperature on the performance of titanium mesh-based MEA for DMFC is analyzed, which provides a technological reference for the preparation of MEA; thus, the influence of test environmental conditions on the performance of titanium mesh-based MEA is explored.

## 2. Materials and Methods

### 2.1. Materials

Carbon paper is brittle, and carbon cloth is difficult to form, while the metal mesh is easy to form, and titanium material has excellent physical and chemical stability. Finally, diamond-shaped titanium mesh is used as an MEA substrate. PtRu/XC-72R (Produced by Johnson Matthey, London, UK) and Pt/XC-72R (Produced by Johnson Matthey, London, UK) are used as MEA anode and cathode catalyst. Vulcan XC-72 carbon (Produced by Cabot, Boston, MA, USA) and polytetrafluoroethylene (PTFE) are selected as the gas diffusion layer of MEA, and the Nafion 117 membrane (Produced by Dupont, Wilmington, DE, USA) is selected as MEA proton exchange membrane. The detailed experimental materials are shown in Table 1.

### 2.2. Preparation Methods of Titanium Mesh-Based MEA

**Figure 2:**
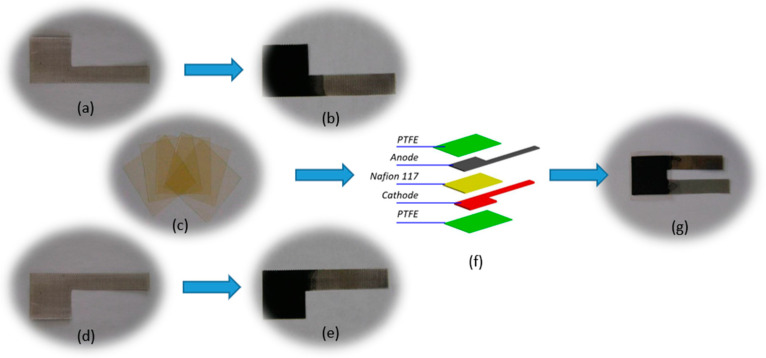
MEA main preparation process photo. (**a**) Titanium mesh substrate; (**b**) titanium mesh-based MEA anode; (**c**) Nafion 117 membrane; (**d**) titanium mesh substrate; (**e**) titanium mesh-based MEA cathode; (**f**) schematic diagram of superposition of titanium mesh-based MEA hot-pressing process; (**g**) titanium mesh-based MEA.

The preparation of titanium mesh-based MEA mainly includes titanium mesh pretreatment [26,27], Nafion 117 membrane pretreatment [28], cathode preparation [18], anode preparation [18], and MEA hot-pressing [13,14,15]. A typical process flow photo is shown in Figure 2.

#### 2.2.1. Titanium Mesh Pretreatment

First, the titanium mesh is treated with 0.5 mol·L^−1^ sulfuric acid solution at 80 °C for 30 min; then, it is ultrasonically cleaned twice with deionized water for 30 min each. Then, the treated titanium mesh is placed in deionized water for use. This process mainly removes impurities on the surface of the titanium mesh. Figure 2a,d show the photos of the prepared electrode-supported titanium mesh.

#### 2.2.2. Nafion 117 Membrane Pretreatment

First, the Nafion 117 membrane is placed in 3% H_2_O_2_ solution and boiled for 30 min; then, it is ultrasonically washed twice with deionized water for 30 min each. Then, it is placed into 1 mol·L^−1^ sulfuric acid, boiled for 60 min, and ultrasonically cleaned with deionized water twice for 30 min each; finally, the treated Nafion 117 membrane is placed into deionized water for use. Figure 2c shows the photo of the Nafion 117 film before pretreatment. Before pretreatment, the film is pale yellow; after pretreatment, it becomes a colorless and transparent body.

#### 2.2.3. Cathode Preparation

A rubber tip dropper is used to absorb the slurry prepared by XC-72 (Cabot, Boston, MA, USA), PTFE emulsion (60 wt.%), and anhydrous ethanol (pure PTFE:XC-72 = 1:1, wt.%). It is dropped onto the surface of the titanium mesh and naturally dried in the air until the load of XC-72C+PTFE reaches 7 mg·cm^−2^. It is then placed in a tube furnace, heat-treated at 340 °C for 30 min under nitrogen protection, and cooled naturally with the furnace to obtain a gas diffusion layer. The cathode catalyst layer is prepared on the surface of the gas diffusion layer by the same drop-coating process as the preparation of the gas diffusion layer. The catalyst slurry is composed of Pt/XC-72R (40 wt.%Pt, Johnson Matthey, London, UK ), Nafion solution (5 wt.%, Dupont, Wilmington, DE, USA), and anhydrous ethanol (pure Nafion:Pt/XC-72R = 1:3, wt.%). After repeated dip coating and drying until the Pt loading in the catalytic layer reaches the requirement of 3 mg·cm^−2^, the titanium mesh-based cathode is prepared, as shown in Figure 2e.

#### 2.2.4. Anode Preparation

The preparation method of the anode catalyst layer is the same as that of the cathode catalyst layer. The difference is that the anode catalyst slurry is composed of PtRu/XC-72R (40 wt.%Pt, 20 wt.%Ru, Johnson Matthey, London, UK), Nafion solution (5 wt.%, Dupont, Wilmington, DE, USA), and anhydrous ethanol (pure Nafion: PtRu/XC-72R = 7:3, wt.%). The final PtRu loading reaches the requirement of 4 mg·cm^−2^. The titanium mesh-based anode is shown in Figure 2d.

#### 2.2.5. MEA Hot-Pressing

The prepared cathode, anode, and pretreated Nafion 117 membrane are stacked in turn to ensure that the cathode catalyst layer and the anode catalyst layer face the Nafion 117 membrane. Then, the PTFE films are superimposed on both sides of the superimposed three-in-one titanium mesh-based MEA as protective films for the hot-pressing titanium mesh-based MEA. Then, a precision press with a heating function is used to prepare the titanium mesh-based MEA by hot-pressing with preset hot-pressing parameters. As the hot-pressing is completed, the PTFE protective film on both sides of the three-in-one titanium mesh-based MEA is removed after the temperature of the titanium mesh-based MEA is cooled. Thus far, the hot-pressing preparation of the titanium mesh-based MEA is completed. Figure 2f shows a schematic diagram of the stacking of the layers of the titanium mesh-based MEA before hot-pressing. Figure 2g shows the photo of the titanium mesh-based MEA with the PTFE protective film removed from the surface.

To study the effect of hot-pressing pressure conditions on the properties of titanium mesh-based MEA, five kinds of titanium mesh-based MEAs with hot-pressing pressure conditions of 0 MPa, 2.5 MPa, 5 MPa, 7.5 MPa, and 10 MPa are prepared by the hot-pressing method. The temperature and pressure holding time are respectively 135 °C and 180 s. To study the effect of hot-pressing temperature on the properties of titanium mesh-based MEA, three kinds of titanium mesh-based MEAs with hot-pressing temperature conditions of 115 °C, 135 °C, and 155 °C are prepared by hot-pressing. The hot-pressing pressure and pressure holding time are 5 MPa and 180 s.

### 2.3. Performance Testing Device and Characterization Methods

**Figure 3:**
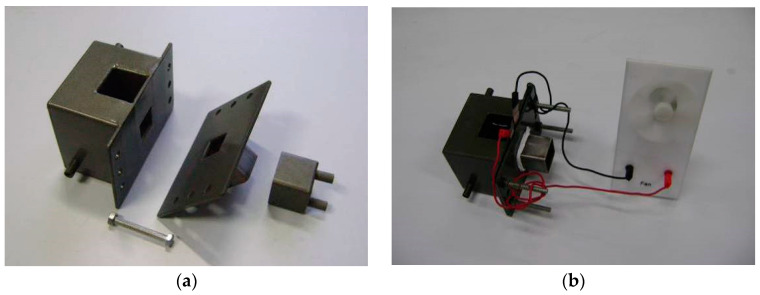
Self-made MEA performance evaluation device and test photos. (**a**) Self-made MEA performance evaluation device; (**b**) photo of MEA driven fan.

The performance testing system of titanium mesh-based MEA consists of a microcomputer, fuel cell test bench, electrochemical workstation, electronic load, super constant temperature water tank, oxygen supply device, and self-made MEA performance evaluation device [29]. To characterize the performance of the titanium mesh-based MEA, a self-made MEA performance testing device is constructed. The main structure is divided into three parts: the anode side, cathode side, and ventilation. It is characterized in that a matching working hole with a size of 20 mm × 20 mm is opened on the sidewall of the anode side and the cathode side facing each other, and bolt holes are opened on the front and rear sides. The ventilation part can be tightly sleeved on the outside of the anode, and oxygen can be introduced through two ventilation pipes. When working, place a silicone gasket or a PTFE gasket on both sides of the cathode and anode of the titanium mesh-based MEA, sandwich it between the cathode side and the anode side the self-made MEA performance evaluation device, and lock it with bolts. Methanol-sulfuric acid electrolyte is injected into the fuel tank on the anode side, and the water bath channel on the anode side can be filled with constant temperature hot water to heat the anode side; oxygen is introduced into the cathode side, or the ventilation device is unplugged to realize air self-breathing. Figure 3 shows the self-made MEA performance testing device and the photo of using the device to test the fan driven by the titanium mesh-based MEA under air self-breathing at room temperature. 

The primary characterization methods include: using the M2D-AT two-dimensional image measuring instrument produced in Shanghai to characterize the micro-morphology of the metal titanium mesh used to prepare the electrode support, the cathode gas diffusion layer and the catalytic layer, and the anode catalytic layer; using a HITACHI S-3400N (Tokyo, Japan) scanning electron microscope for SEM and EDX analysis of titanium mesh-based MEA; using the LSV function of Shanghai Chenhua CHI660C (Shanghai, China) electrochemical workstation to test the polarization performance of MEA. The scanning voltage frequency is 1 mV·s^−1^, the initial potential is the open-circuit voltage of the titanium mesh-based MEA, the termination potential is 0 V, and the sampling interval is 1 s [30,31].

The activation process of the titanium mesh-based MEA: first, the titanium mesh-based MEA is placed in deionized water for 60 min at 60 °C, and then put in the self-made evaluation device. A total of 1 mol·L^−1^ methanol (Shanghai Chemical Reagent Co., Ltd., Shanghai, China) and 0.5 mol·L^−1^ sulfuric acid (Shanghai Chemical Reagent Co., Ltd., Shanghai, China) electrolyte is added to the anode side, and 100 mL·min^−1^ oxygen at 0.1 MPa is introduced to the cathode side. MEA is heated in a circulating water bath and activated at 60 °C for 120 min. Then, it is continuously discharged with a low-power (20 mW) electric fan for 5 min to 10 min. Finally, it is cooled to room temperature to complete the activation process of the titanium mesh-based MEA [26,27].

## 3. Results

### 3.1. Morphology Observation and Elemental Analysis of Electrode

#### 3.1.1. Micro Morphology of Electrode Preparation Process

**Figure 4:**
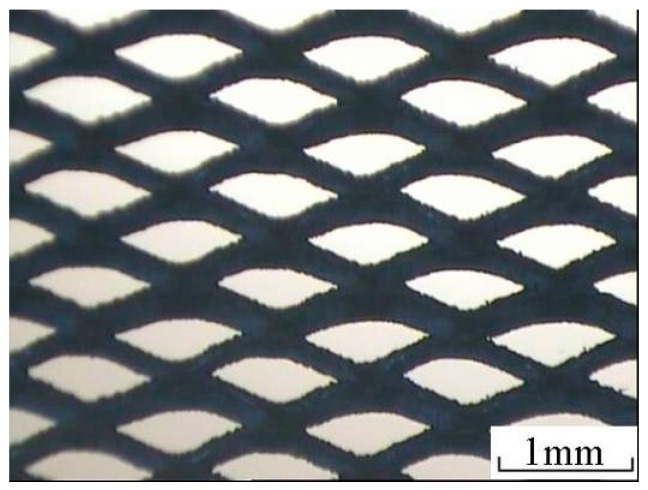
Micro morphology of electrode substrate MEA.

The thickness of the titanium mesh used to prepare MEA is 250 μm. The mesh size is 0.5 mm × 1.2 mm, the porosity is about 43%, and the shape of the titanium mesh is shown in Figure 4.

**Figure 5:**
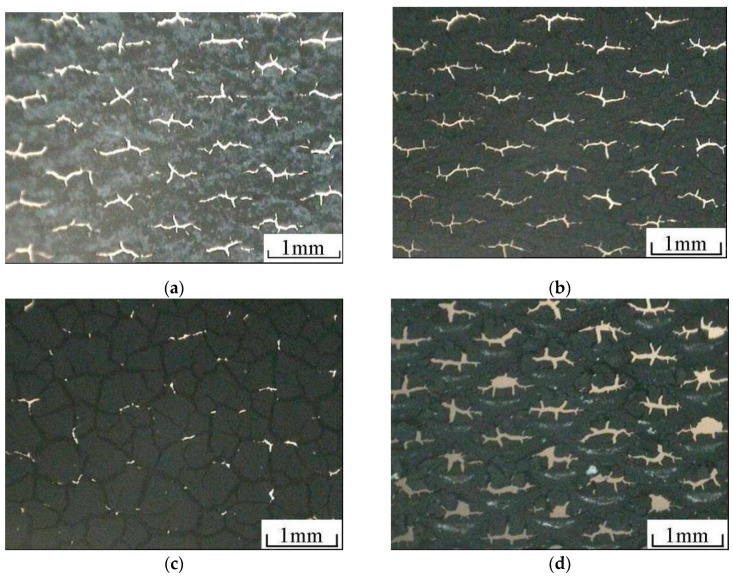
Micro morphology of titanium mesh-based MEA: (**a**) Cathode gas diffusion layer before heat treatment; (**b**) cathode gas diffusion layer after heat treatment; (**c**) cathode catalytic layer; (**d**) anode catalytic layer.

Figure 5a–d shows the micrographs of the surface of the cathode gas diffusion layer before the heat treatment, cathode gas diffusion layer after the heat treatment, cathode catalytic layer, and anode catalytic layer.

As shown in Figure 5a, white PTFE material is evenly distributed on the surface of the cathode gas diffusion layer, and the hole of titanium mesh still exists. Figure 5b shows that, after 340 °C heat treatment, the white PTFE on the surface of the gas diffusion layer melts and blends evenly with XC-72 carbon in the gas diffusion layer, which is conducive to better bonding between the gas diffusion layer and the titanium mesh substrate, so the surface presents uniform black. It can be observed from Figure 5c that there are micro cracks on the surface of the cathode catalytic layer, but the diamond mesh of the titanium mesh is covered. As the Nafion solution is added into the catalyst slurry as the binder and a more reasonable coating is adopted, it is conducive to the coating of small particle catalyst on the surface of the cathode gas diffusion layer. It improves the bonding ability between the catalyst and the gas diffusion layer. Figure 5d shows that there are also microcracks on the surface of the anode catalytic layer. Similarly, the Nafion solution is added to the catalyst slurry as the binder, and a more reasonable coating process is adopted, which is conducive to the coating of a small particle catalyst on the surface of the anode titanium mesh and improves the bonding ability between the catalyst and the titanium mesh [15]. Although the prepared cathode and anode still have microcracks, in the later single cell performance test, there is no catalyst-shedding phenomenon. The performance is also relatively stable when used many times. In addition, the existence of microcracks is also conducive to improving the catalytic effect of the catalyst in the electrode reaction [32,33].

#### 3.1.2. Micro Morphology of Electrode Section

Table 2 shows the titanium mesh’s forming thickness and compressibility data based on MEA under different hot-pressing pressures. Sample 1 is the thickness of the cathode, with the Nafion 117 membrane and anode naturally close together. Its thickness is the maximum thickness of all samples and the thickness of other samples before hot pressing. With the increase in pressure, the thickness of the titanium mesh-based MEA gradually becomes thinner. The material’s compressibility gradually increases, which will inevitably lead to the decrease in porosity in MEA. When the pressure increases from 0 MPa to 2.5 MPa, the thickness of the titanium mesh-based MEA increases from 850 μm and decreases to 690 μm. This is due to the loose material deposited on the surface of the electrode support titanium mesh prepared by the drop-coating process of the cathode and anode; when the pressure increases from 2.5 MPa to 10 MPa, the forming thickness of titanium mesh-based MEA shows a linear change. For each increase of 2.5 MPa, the thickness of titanium mesh-based MEA becomes thinner by 30 μm [13].

**Figure 6:**
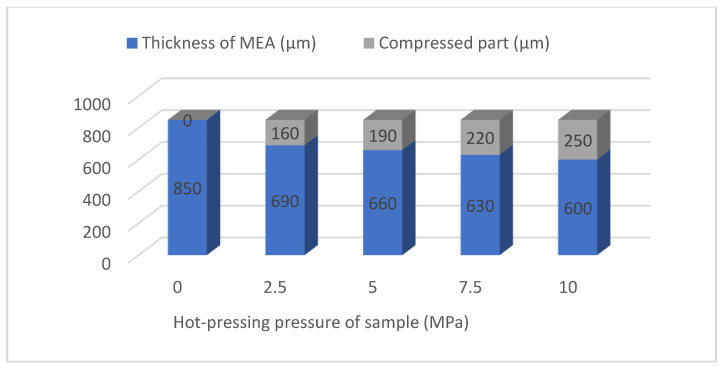
The influence of different forming pressure conditions on the thickness and compressibility of titanium mesh-based MEA.

Figure 6 shows more intuitive results of the influence of different forming pressure conditions on the thickness and compressibility of titanium mesh-based MEA.

**Figure 7:**
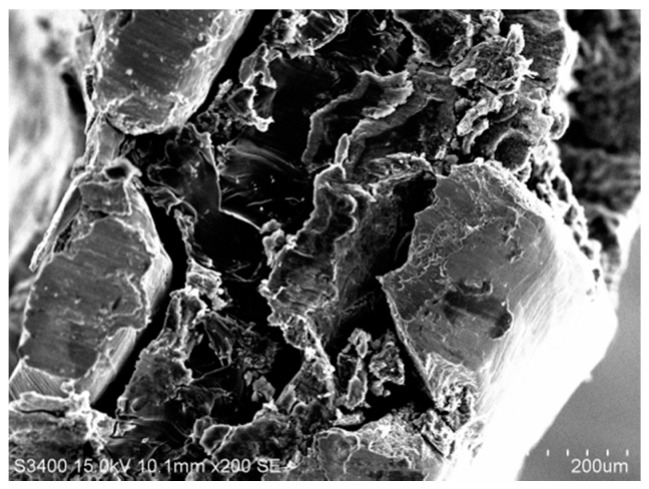
SEM analysis of the cross-section of titanium mesh-based MEA.

Figure 7 shows the SEM analysis of the cross-section of titanium mesh-based MEA with a hot-pressing pressure of 7.5 MPa, a hot-pressing temperature of 135 °C, and a hot-pressing time of 180 s, which demonstrates that the silk stem of the titanium mesh is prominent. However, the stratification of the cathode gas diffusion layer, cathode catalytic layer, Nafion 117 membrane, and anode catalytic layer is not very obvious. This is mainly due to a too-high forming pressure, resulting in the cathode gas diffusion layer, cathode catalytic layer, Nafion 117 membrane, and anode catalytic layer being severely deformed by the titanium mesh extrusion, which is a distorted layer in the figure.

#### 3.1.3. Element Analysis of Electrode Cross-Section

**Figure 8:**
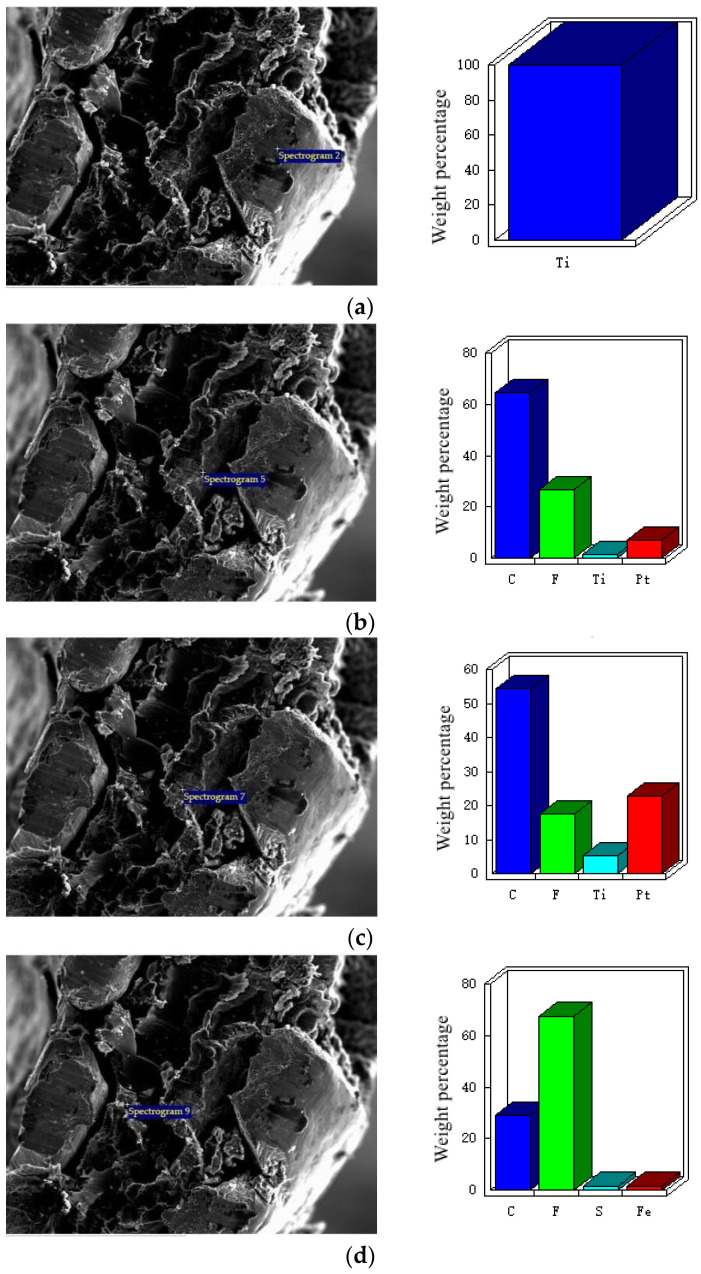

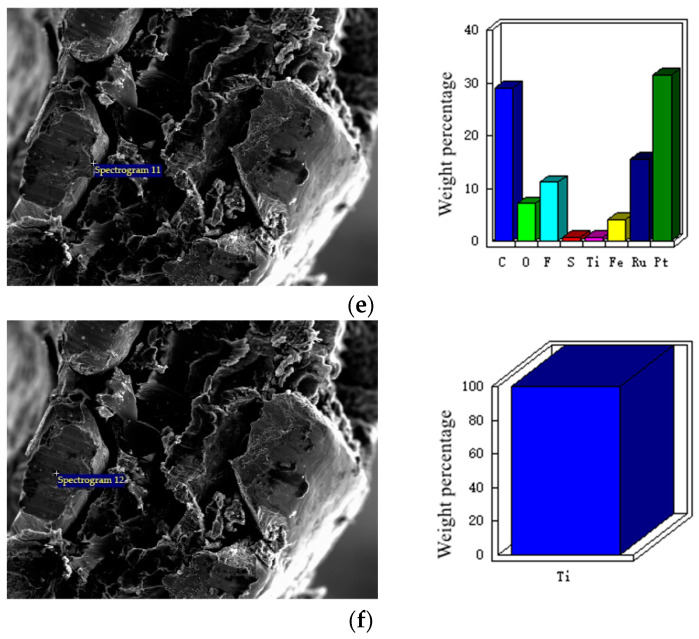
EDX analysis diagram of each layer in the cross-section of titanium mesh-based MEA. (**a**) Cathode substrate; (**b**) cathode gas diffusion layer; (**c**) cathode catalytic layer; (**d**) Nafion 117 membrane; (**e**) anode catalytic layer; (**f**) anode substrate.

Figure 8 is the EDX analysis diagram of each layer in the cross-section of titanium mesh-based MEA. It can be observed from the figure that the EDX analysis results of each point are consistent with the reagent formula of each layer. Figure 8a,f show the EDX point analysis of cathode substrate and anode substrate, respectively, whose results show that the weight percentage of Ti element reaches 100%. Figure 8b shows the EDX point analysis of the cathode gas diffusion layer, whose results demonstrate that it mainly contains the elements C and F, which are the elements contained in the two significant reagents, XC-72 carbon and PTFE, of the catalytic layer. At the same time, it contains a small amount of Pt, which indicates that the cathode catalyst and the gas diffusion layer are fused, which is caused by the existence of holes in the gas diffusion layer in Figure 5b above. Figure 8c shows the EDX point analysis of the cathode catalytic layer, whose results demonstrate that it mainly contains C, F, and Pt elements. F is the element introduced into the cathode gas diffusion layer, and Pt is the main element of the cathode catalyst. Figure 8d shows the EDX point analysis of the Nafion 117 membrane, whose results demonstrate that it mainly contains C, F, and S elements, which is consistent with the material properties. Figure 8e shows the EDX point analysis of the anode catalytic layer, whose results show that the weight percentage of C, Pt, and Ru elements is the same as that in the anode catalyst, PtRu/XC-72R. At the same time, it also shows that there are F and O elements, indicating that the anode catalyst has been partially embedded in the Nafion 117 membrane [31,34].

### 3.2. Effect of Hot-Pressing Pressure on the Performance of Titanium Mesh-Based MEA

Under the hot-pressing time of 180 s and hot-pressing temperature of 135 °C, the effects of five hot-pressing pressure conditions of 0 MPa, 2.5 MPa, 5 MPa, 7.5 MPa, and 10 MPa on the performance of titanium mesh-based MEA were studied. The result was given as follows.

#### 3.2.1. Performance at Room Temperature with Air Self-Breathing Environment

**Figure 9:**
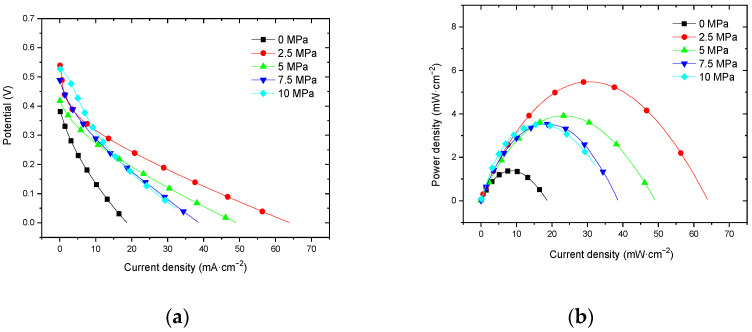
The effect of hot-pressing pressure on the performance of titanium mesh-based MEA at room temperature with air self-breathing environment. (**a**) Curve between current density and voltage; (**b**) curve between current density and power density.

Figure 9 shows the curve of the effect of hot-pressing pressure on the performance of titanium mesh-based MEA at room temperature (25 °C) and atmospheric pressure with air self-breathing as the cathode oxidant and 1 mol·L^−1^ methanol + 0.5 mol·L^−1^ sulfuric acid aqueous solution as the anode electrolyte. It can be observed from the figure that the peak power density of titanium mesh-based MEA with a hot-pressing pressure of 2.5 MPa is the largest. Its maximum value is 5.47 mW·cm^−2^, which is 3.96 times the MEA peak power density of 1.38 mW·cm^−2^ under a 0 MPa hot-pressing pressure, 1.40 times the MEA peak power density of 3.91 mW·cm^−2^ under a 5 MPa hot-pressing pressure, 1.55 times the MEA peak power density of 3.53 mW·cm^−2^ under a 7.5 MPa hot-pressing pressure, and 1.55 times the MEA peak power density of 3.52 mW·cm^−2^ under a 10 MPa hot-pressing pressure.

#### 3.2.2. Performance at Room Temperature with Oxygen Environment

**Figure 10:**
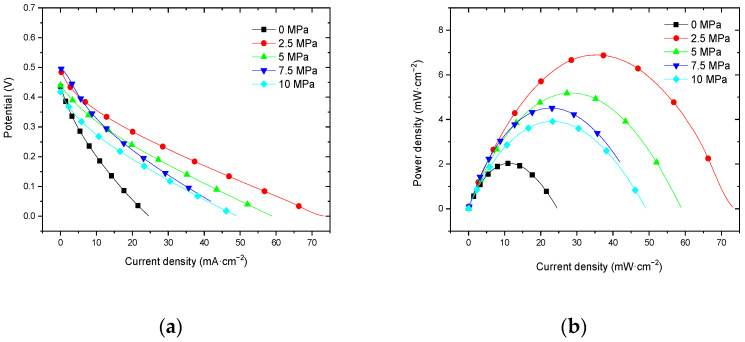
The effect of hot-pressing pressure on the performance of titanium mesh-based MEA at room temperature with an oxygen environment. (**a**) Curve between current density and voltage; (**b**) curve between current density and power density.

Figure 10 shows the curve of the effect of hot-pressing pressure on the performance of titanium mesh-based MEA at room temperature (25 °C) and atmospheric pressure with 100 mL·min^−1^ oxygen of 0.1 MPa as the cathode oxidant and 1 mol·L^−1^ methanol + 0.5 mol·L^−1^ sulfuric acid aqueous solution as the anode electrolyte. It can be observed from the figure that the peak power density of titanium mesh-based MEA with a hot-pressing pressure of 2.5 MPa is the largest. Its maximum value is 6.89 mW·cm^−2^, which is 3.38 times the MEA peak power density of 2.04 mW·cm^−2^ under a 0 MPa hot-pressing pressure, 1.33 times of the MEA peak power density of 5.19 mW·cm^−2^ under a 5 MPa hot-pressing pressure, 1.53 times of the MEA peak power density of 4.51 mW·cm^−2^ under a 7.5 MPa hot-pressing pressure, and 1.76 times the MEA peak power density of 3.91 mW·cm^−2^ under a 10 MPa hot-pressing pressure.

#### 3.2.3. Performance at 60 °C with Air Self-Breathing Environment

**Figure 11:**
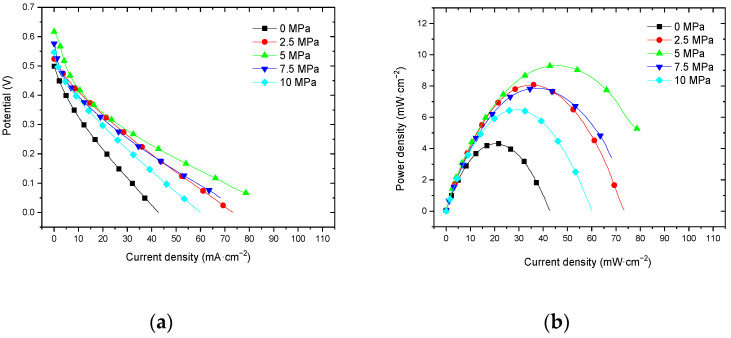
The effect of hot-pressing pressure on the performance of titanium mesh-based MEA at 60 °C with air self-breathing environment. (**a**) Curve between current density and voltage; (**b**) curve between current density and power density.

Figure 11 shows the curve of the effect of hot-pressing pressure on the performance of titanium mesh-based MEA at 60 °C and atmospheric pressure with 100 mL·min^−1^ oxygen as the cathode oxidant and a 1 mol·L^−1^ methanol + 0.5 mol·L^−1^ sulfuric acid aqueous solution as the anode electrolyte. It can be observed from the figure that the peak power density of the titanium mesh-based MEA with a hot-pressing pressure of 5 MPa is the largest. Its maximum value is 9.31 mW·cm^−2^, which is 2.15 times the MEA peak power density of 4.32 mW·cm^−2^ under a 0 MPa hot-pressing pressure, 1.15 times the MEA peak power density of 8.08 mW·cm^−2^ under a 2.5 MPa hot-pressing pressure, 1.18 times the MEA peak power density of 7.86 mW·cm^−2^ under a 7.5 MPa hot-pressing pressure, and 1.43 times the MEA peak power density of 6.50 mW·cm^−2^ under a 10 MPa hot-pressing pressure.

#### 3.2.4. Performance at 60 °C with Oxygen Environment

**Figure 12:**
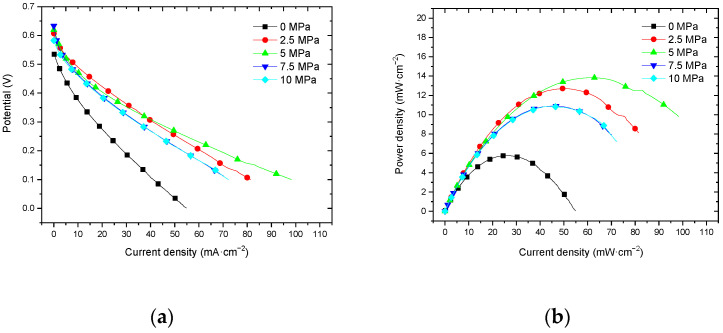
The effect of hot-pressing pressure on the performance of titanium mesh-based MEA at 60 °C with an oxygen environment. (**a**) Curve between current density and voltage; (**b**) curve between current density and power density.

Figure 12 shows the curve of the effect of the hot-pressing pressure on the performance of titanium mesh-based MEA at 60 °C and atmospheric pressure with air self-breathing as the cathode oxidant and a 1 mol·L^−1^ methanol + 0.5 mol·L^−1^ sulfuric acid aqueous solution as the anode electrolyte. It can be observed from the figure that the peak power density of titanium mesh-based MEA with a hot-pressing pressure of 5 MPa is the largest. Its maximum value is 13.83 mW·cm^−2^, which is 2.39 times the MEA peak power density of 5.79 mW·cm^−2^ under a 0 MPa hot-pressing pressure, 1.09 times the MEA peak power density of 12.72 mW·cm^−2^ under 2.5 MPa hot-pressing pressure, 1.26 times the MEA peak power density of 10.90 mW·cm^−2^ under a 7.5 MPa hot-pressing pressure, and 1.27 times the MEA peak power density of 10.85 mW·cm^−2^ under a 10 MPa hot-pressing pressure.

### 3.3. Effect of Hot-Pressing Temperature on the Performance of Titanium Mesh-Based MEA

Under the conditions of the hot-pressing time of 180 s and hot-pressing pressure of 5 MPa, the effects of three hot-pressing temperature conditions of 115 °C, 135 °C, and 155 °C on the performance of MEA are studied. The result is given as follows.

#### 3.3.1. Performance at Room Temperature with Air Self-Breathing Environment

**Figure 13:**
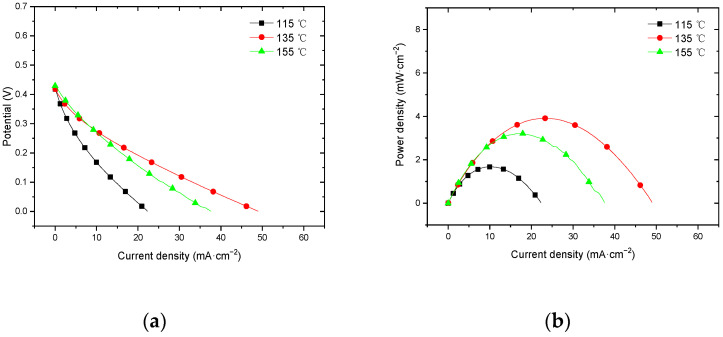
The effect of hot-pressing temperature on the performance of titanium mesh-based MEA at room temperature with air self-breathing environment. (**a**) Curve between current density and voltage; (**b**) curve between current density and power density.

Figure 13 shows the curve of the effect of hot-pressing temperature on the performance of titanium mesh-based MEA at room temperature (25 °C) and atmospheric pressure with air self-breathing as the cathode oxidant and a 1 mol·L^−1^ methanol + 0.5 mol·L^−1^ sulfuric acid aqueous solution as the anode electrolyte. It can be observed from the figure that the peak power density of the titanium mesh-based MEA with the hot-pressing temperature of 135 °C is the largest. Its maximum value is 3.91 mW·cm^−2^, which is 2.77 times the MEA peak power density of 1.41 mW·cm^−2^ at 115 °C and 1.22 times the MEA peak power density of 3.21 mW·cm^−2^ at 155 °C.

#### 3.3.2. Performance at Room Temperature with Oxygen Environment

**Figure 14:**
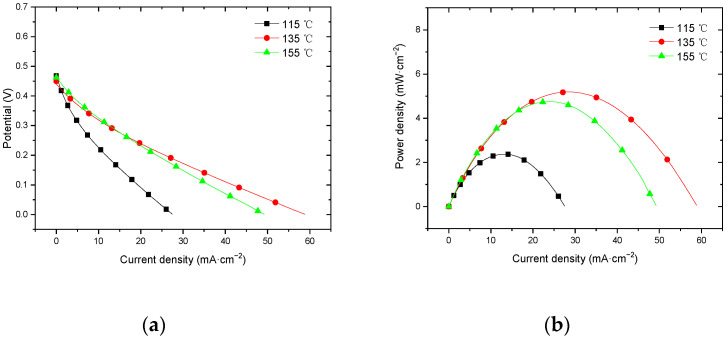
The effect of hot-pressing temperature on the performance of titanium mesh-based MEA at room temperature with oxygen environment. (**a**) Curve between current density and voltage; (**b**) curve between current density and power density.

Figure 14 shows the curve of the effect of hot-pressing temperature on the performance of titanium mesh-based MEA at room temperature (25 °C) and atmospheric pressure with 100 mL·min^−1^ oxygen of 0.1 MPa as the cathode oxidant and a 1 mol·L^−1^ methanol + 0.5 mol·L^−1^ sulfuric acid aqueous solution as the anode electrolyte. It can be observed from the figure that the peak power density of titanium mesh-based MEA with the hot-pressing temperature of 135 °C is the largest. Its maximum value is 5.19 mW·cm^−2^, which is 1.54 times the MEA peak power density of 3.38 mW·cm^−2^ at 115 °C and 1.09 times the MEA peak power density of 4.75 mW·cm^−2^ at 155 °C.

#### 3.3.3. Performance at 60 °C with Air Self-Breathing Environment

**Figure 15:**
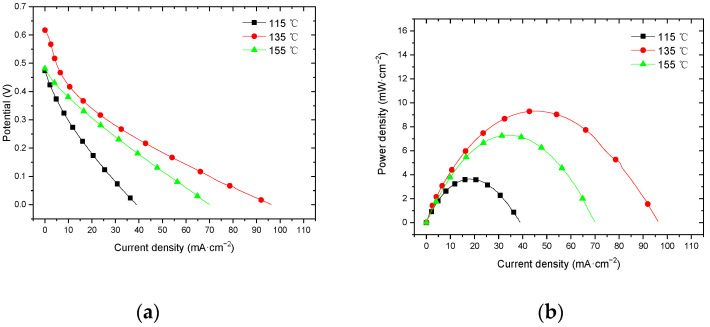
The effect of hot-pressing temperature on the performance of titanium mesh-based MEA at room temperature with oxygen environment. (**a**) Curve between current density and voltage; (**b**) curve between current density and power density.

Figure 15 shows the curve of the effect of hot-pressing temperature on the performance of titanium mesh-based MEA at 60 °C and atmospheric pressure with air self-breathing as the cathode oxidant and a 1 mol·L^−1^ methanol + 0.5 mol·L^−1^ sulfuric acid aqueous solution as the anode electrolyte. 

It can be observed from the figure that the peak power density of titanium mesh-based MEA with a hot-pressing temperature of 135 °C is the largest, and its maximum value is 9.31 mW·cm^−2^, which is 2.55 times the MEA peak power density of 3.65 mW·cm^−2^ at 115 °C and 1.28 times the MEA peak power density of 7.30 mW·cm^−2^ at 155 °C.

#### 3.3.4. Performance at 60 °C with Oxygen Environment

**Figure 16:**
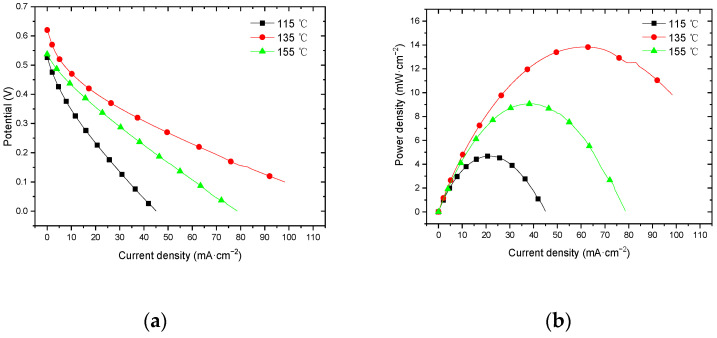
The effect of hot-pressing temperature on the performance of titanium mesh-based MEA at 60 °C with an oxygen environment. (**a**) Curve between current density and voltage; (**b**) curve between current density and power density.

Figure 16 shows the curve of the effect of the hot-pressing temperature on the performance of titanium mesh-based MEA at 25 °C and atmospheric pressure with 100 mL·min^−1^ oxygen of 0.1 MPa as the cathode oxidant and a 1 mol·L^−1^ methanol + 0.5 mol·L^−1^ sulfuric acid aqueous solution as the anode electrolyte. It can be observed from the figure that the peak power density of titanium mesh-based MEA with the hot-pressing temperature of 135 °C is the largest. Its maximum value is 13.83 mW·cm^−2^, which is 2.96 times the MEA peak power density of 4.68 mW·cm^−2^ at 115 °C and 1.53 times the MEA peak power density of 9.06 mW·cm^−2^ at 155 °C.

## 4. Discussion

### 4.1. Influence of Hot-Pressing Pressure on the Forming Thickness of MEA

#### 4.1.1. Linear Fitting Analysis with Full Parameters

**Figure 17:**
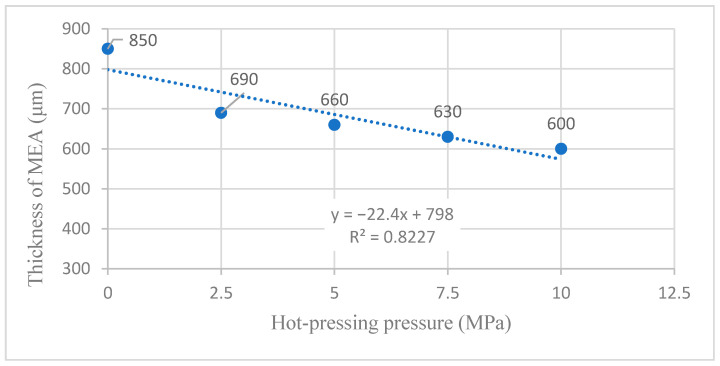
The fitting curve of the thickness of MEA at different hot-pressing pressures.

According to the experimental data in Table 2 and the linear fitting method [35], the linear fitting between the forming thickness of MEA and the hot-pressing pressure is performed to obtain the fitting diagram and linear formula as shown in Figure 17 and Equation (1):

(1) y=−22.4x+798

(2) $$R^{2}=0.8227$$

where y refers to the forming thickness of MEA and x refers to the hot-pressing pressure. $R^{2}$ is the coefficient of determination.

#### 4.1.2. Linear Fitting Analysis without 0 MPa Sample

**Figure 18:**
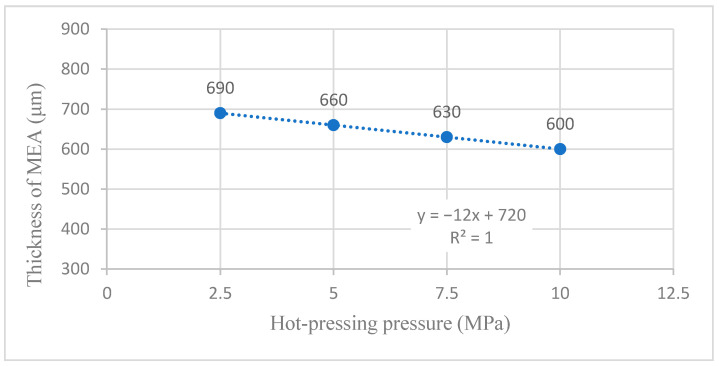
The fitting curve of the thickness of MEA at different hot-pressing pressure without 0 MPa sample.

According to the results in Section 3.1.2, Sample 1 is the thickness of the cathode, Nafion 117 membrane, and anode, which are naturally close together. Its thickness is the maximum thickness of all samples and the thickness of other samples before hot pressing. Of course, this thickness parameter also has some randomness. When the thickness of the 0 MPa sample is ignored, the linear fitting between the forming thickness of MEA and the hot-pressing pressure is performed to obtain the fitting diagram and linear formula, as shown in Figure 18 and Equation (3):

(3) y=−12x+720

(4) $$R^{2}=1$$

where y refers to the forming thickness of MEA and x refers to the hot-pressing pressure. $R^{2}$ is the coefficient of determination, which changed from 0.8227 to 1. Therefore, the fitting graph’s linear Formula (3) becomes a simple linear formula. Again, the pressure control of the precision press is very linear, which provides a reasonable basis for the accurate preparation of the samples [13].

### 4.2. Influence of Hot-Pressing Pressure on the Peak Power Density

#### 4.2.1. Performance Comparison at Room Temperature

**Figure 19:**
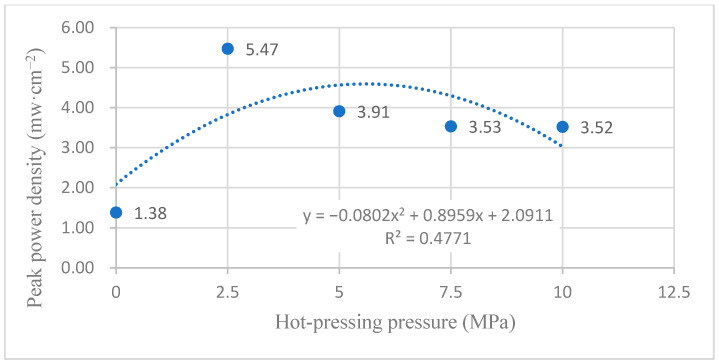
The fitting curve of the peak power density of MEA at different hot-pressing pressures under room temperature and air conditions.

According to the experimental data of Figure 9 and the polynomial fitting method [36], the fitting between the peak power density and the hot-pressing pressure under room temperature and air conditions is performed to obtain the fitting diagram and polynomial formula, as shown in Figure 19 and Equation (5):

(5) $$y=-0.0802x^{2}+0.8959x+2.0911$$

(6) $$R^{2}=0.4771$$

where y refers to the thickness of MEA and x refers to the hot-pressing pressure. $R^{2}$ is the coefficient of determination.

**Figure 20:**
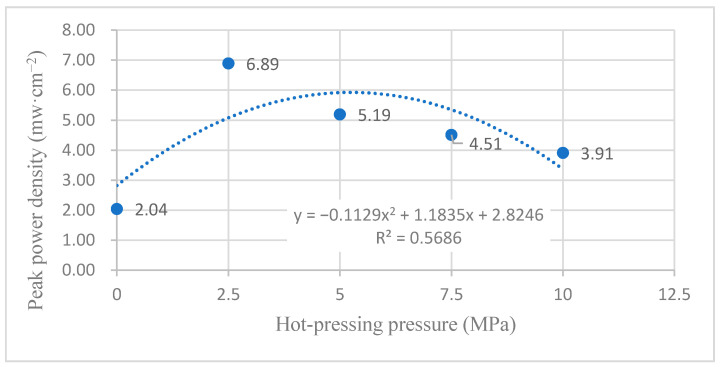
The fitting curve of the peak power density of MEA at different hot-pressing pressures under room temperature and oxygen conditions.

According to the experimental data in Figure 10, the fitting between the peak power density and the hot-pressing pressure under room temperature and oxygen conditions is performed to obtain the fitting diagram and polynomial formula, as shown in Figure 20 and Equation (7):

(7) $$y=-0.1129x^{2}+1.1835x+2.8246$$

(8) $$R^{2}=0.5686$$

where y refers to the thickness of MEA and x refers to the hot-pressing pressure. $R^{2}$ is the coefficient of determination.

Comparing Figure 9, Figure 10, Figure 19 and Figure 20, it can be found that under room temperature, whether the cathode oxidant is self-breathing air or 100 mL·min^−1^ oxygen of 0.1 MPa, the influence of hot-pressing pressure on the polarization performance of titanium mesh-based MEA shows a consistent regularity. That is, when the hot-pressing pressure gradually increases from 0 MPa to 2.5 MPa, the peak power density of titanium mesh-based MEA gradually increases. When the hot-pressing pressure gradually increases from 2.5 MPa to 10 MPa, the peak power density of titanium mesh-based MEA gradually decreases. The results demonstrate that the titanium mesh-based MEA with a hot-pressing pressure of 2.5 MPa at room temperature is better than that of titanium mesh-based MEA under other hot-pressing pressure conditions. In addition, the performance of the titanium mesh-based MEA is better than the data reported in the latest literature [37], whose peak power density is 1.67 mW·cm^−2^ at room temperature and air-breathing.

#### 4.2.2. Performance Comparison at 60 °C

**Figure 21:**
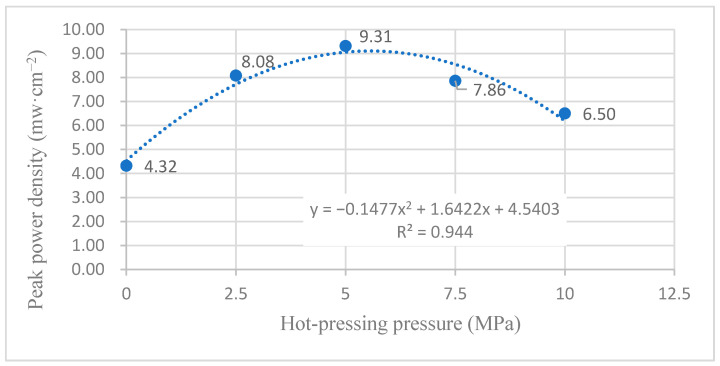
The fitting curve of the peak power density of MEA at different hot-pressing pressures under 60 °C and air conditions.

According to the experimental data in Figure 11, the fitting between the peak power density and the hot-pressing pressure under 60 °C and air conditions is performed to obtain the fitting diagram and polynomial formula, as shown in Figure 21 and Equation (9):

(9) $$y=-0.1477x^{2}+1.6422x+4.5403$$

(10) $$R^{2}=0.944$$

where y refers to the thickness of MEA and x refers to the hot-pressing pressure. $R^{2}$ is the coefficient of determination.

**Figure 22:**
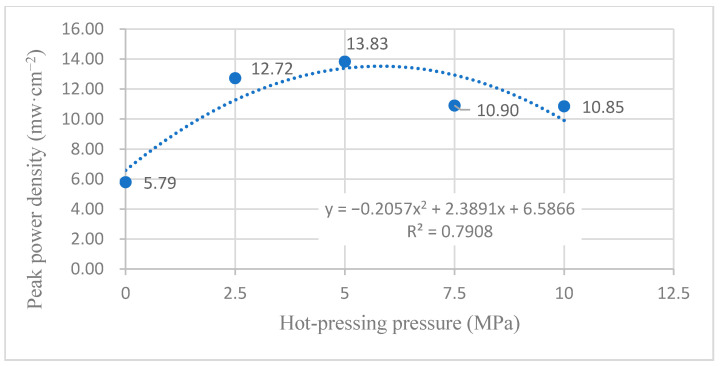
The fitting curve of the peak power density of MEA at different hot-pressing pressures under 60 °C and oxygen conditions.

According to the experimental data of Figure 12, the fitting between the peak power density and the hot-pressing pressure under 60 °C and oxygen conditions is performed to obtain the fitting diagram and polynomial formula, as shown in Figure 22 and Equation (11):

(11) $$y=-0.2057x^{2}+2.3891x+6.5866$$

(12) $$R^{2}=0.7908$$

where y refers to the thickness of MEA and x refers to the hot-pressing pressure. $R^{2}$ is the coefficient of determination.

Comparing Figure 11, Figure 12, Figure 21 and Figure 22, it can be found that under 60 °C, whether the cathode oxidant is self-breathing air or 100 mL·min^−1^ oxygen of 0.1 MPa, the influence of hot-pressing pressure on the polarization performance of titanium mesh-based MEA shows a consistent regularity. That is, when the hot-pressing pressure gradually increases from 0 MPa to 2.5 MPa, the peak power density of titanium mesh-based MEA gradually increases. When the hot-pressing pressure gradually increases from 2.5 MPa to 10 MPa, the peak power density of titanium mesh-based MEA gradually decreases. The results demonstrate that the titanium mesh-based MEA with a hot-pressing pressure of 2.5 MPa at 60 °C is better than that of the titanium mesh-based MEA under other hot-pressing pressure conditions.

#### 4.2.3. Comparison of the Comprehensive Properties

According to the comprehensive comparison of Figure 19, Figure 20, Figure 21 and Figure 22, under room temperature and high temperature (60 °C) conditions, the peak value of the maximum power density appears in the titanium mesh-based MEA with hot-pressing pressures of 2.5 MPa and 5 MPa, respectively. In addition, it can be observed from Table 2 in Section 3.1.2 that the compression rate of the titanium mesh-based MEA gradually increases and the porosity of titanium mesh-based MEA correspondingly decreases during the gradual increase in hot-pressing pressure from 0 MPa to 10 MPa; the pore size and porosity of titanium mesh-based MEA are closely related to its mass transfer capacity [13].

When the hot-pressing pressure condition is 0 MPa, it depends on the clamping force of the test device, which cannot ensure the close combination of the three layers, which will produce large impedance and will affect the polarization performance of titanium mesh-based MEA; this is also the main reason for affecting the polarization performance of titanium mesh-based MEA. When the hot-pressing pressure is 2.5 MPa, the cathode, Nafion 117 membrane, and anode can be easily hot pressed as a whole, and the contact impedance is lower than that of titanium mesh-based MEA when the hot-pressing pressure is 0 MPa. At this time, the titanium mesh-based MEA demonstrates better room temperature polarization performance, indicating that the porosity and pore size of titanium mesh-based MEAs are more suitable for the mass transfer at room temperature [17].

With the increase in reaction temperature, the activation energy of both cathode oxidant gas and anode electrolyte will increase, so as to improve the reaction efficiency of anode and cathode. At the same time, large porosity and pore size (such as titanium mesh-based MEA with a hot-pressing pressure of 2.5 MPa) will lead to serious methanol leakage and affect the polarization performance of titanium mesh-based MEA. Therefore, when the hot-pressing pressure is 5 MPa, the titanium mesh-based MEA shows good high-temperature polarization performance, indicating that the porosity and pore size of titanium mesh-based MEA are more suitable for mass transfer at high temperatures [15].

As the forming pressure increases, the compression ratio of titanium mesh-based MEA also gradually increases, resulting in the gradual compression deformation of the Nafion 117 membrane. The methanol leakage phenomenon will also increase. The pore sizes of the cathode and anode will gradually decrease, which is not conducive to the gas diffusion of materials, thus affecting the polarization performance of titanium mesh-based MEA. It is generally considered that the optimum working temperature of DMFC is 60 °C [25]; thus, this paper considers that under the optimum operating temperature of the fuel cell, the optimal forming pressure conditions of the titanium mesh-based MEA is 5 MPa.

### 4.3. Influence of Hot-Pressing Temperature on the Peak Power Density

#### 4.3.1. Performance Comparison at Room Temperature

**Figure 23:**
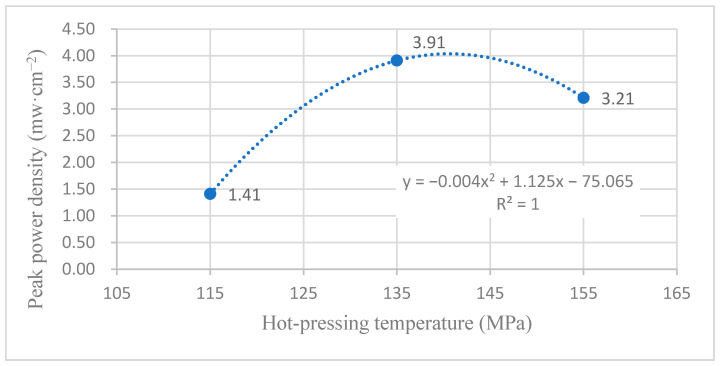
The fitting curve of the peak power density of MEA at a different hot-pressing temperature under room temperature and air conditions.

According to the experimental data of Figure 13 and the polynomial fitting method [36], the fitting between the peak power density and the hot-pressing temperature under room temperature and air conditions is performed to obtain the fitting diagram and polynomial formula, as shown in Figure 23 and Equation (13):

(13) $$y=-0.004x^{2}+1.125x-75.065$$

(14) $$R^{2}=1$$

where y refers to the thickness of MEA and x refers to the hot-pressing pressure. $R^{2}$ is the coefficient of determination.

**Figure 24:**
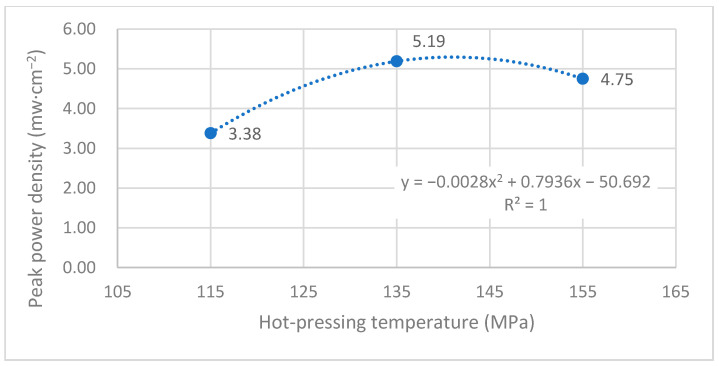
The fitting curve of the peak power density of MEA at a different hot-pressing temperature under room temperature and oxygen conditions.

According to the experimental data in Figure 14, the fitting between the peak power density and the hot-pressing temperature under room temperature and oxygen conditions is performed to obtain the fitting diagram and polynomial formula, as shown in Figure 24 and Equation (15):

(15) $$y=-0.028x^{2}+0.7936x-50.692$$

(16) $$R^{2}=1$$

where y refers to the thickness of MEA and x refers to the hot-pressing pressure. $R^{2}$ is the coefficient of determination.

Comparing Figure 13, Figure 14, Figure 23 and Figure 24, it can be found that the peak power density appears on the titanium mesh-based MEA under the hot-pressing temperature of 135 °C at room temperature. 

#### 4.3.2. Performance Comparison at 60 °C

**Figure 25:**
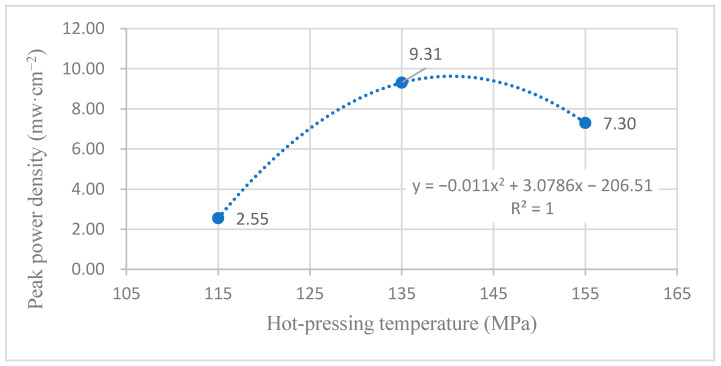
The fitting curve of the peak power density of MEA at a different hot-pressing temperature under 60 °C and air conditions.

According to the experimental data in Figure 15, the fitting between the peak power density and the hot-pressing temperature under 60 °C and air conditions is performed to obtain the fitting diagram and polynomial formula, as shown in Figure 25 and Equation (17):

(17) $$y=-0.011x^{2}+3.0786x-206.51$$

(18) $$R^{2}=1$$

where y refers to the thickness of MEA and x refers to the hot-pressing pressure. $R^{2}$ is the coefficient of determination.

**Figure 26:**
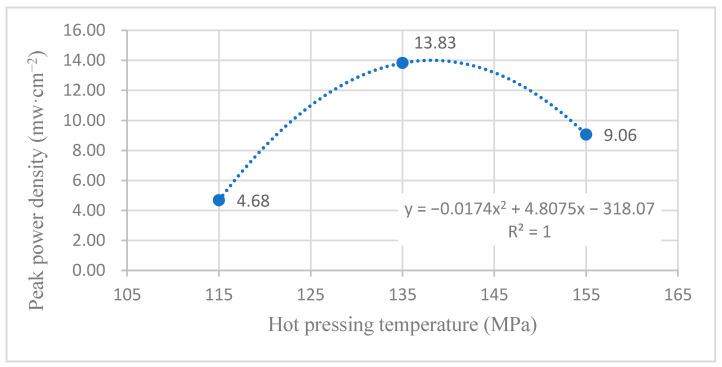
The fitting curve of the peak power density of MEA at a different hot-pressing temperature under 60 °C and oxygen conditions.

According to the experimental data in Figure 15, the fitting between the peak power density and the hot-pressing temperature under 60 °C and oxygen conditions is performed to obtain the fitting diagram and polynomial formula, as shown in Figure 26 and Equation (19):

(19) $$y=-0.0174x^{2}+4.8075x-318.07$$

(20) $$R^{2}=1$$

where y refers to the thickness of MEA and x refers to the hot-pressing pressure. $R^{2}$ is the coefficient of determination.

Comparing Figure 15, Figure 16, Figure 25 and Figure 26, it can be found that the peak power density appears on the titanium mesh-based MEA under the hot-pressing temperature of 135 °C at a high temperature (60 °C). 

#### 4.3.3. Comparison of the Comprehensive Properties

Comprehensively comparing Figure 23, Figure 24, Figure 25 and Figure 26, the peak power density appears on the titanium mesh-based MEA under the hot-pressing temperature of 135 °C at room temperature and high temperature (60 °C). This is because when the hot-pressing temperature rises from 115 °C to 135 °C, the Nafion film becomes softer, and the bonding interface with the catalytic layer becomes more extensive, which reduces the internal resistance of the titanium mesh-based MEA; thus, the titanium mesh-based MEA can obtain higher output power density [17]. When the hot-pressing temperature continues to rise from 135 °C to 155 °C, the loss of sulfonic acid group in the Nafion membrane will gradually increase, affecting the ability of the Nafion membrane to transfer protons, and then affecting the output power density of the titanium mesh-based MEA [33].

## 5. Conclusions

The following conclusions are obtained by studying the effect of the hot-pressing process conditions on the properties of titanium mesh-based MEA for DMFC.

(1) Under the premise of a hot-pressing time of 180 s and the optimal operating temperature of DMFC of 60 °C, the experimental results demonstrate that the appropriate hot-pressing process conditions of titanium mesh-based MEA are a hot-pressing pressure of 5 MPa and a hot-pressing temperature of 135 °C.

(2) When the hot-pressing temperature is 135 °C and the pressure holding time is 180 s, the MEA is prepared with hot-pressing pressures of 0 MPa, 2.5 MPa, 5 MPa, 7.5 MPa, and 10 MPa, respectively, and the forming thickness of titanium mesh MEA is studied. With the increasing forming pressure, the forming thickness of the titanium mesh MEA gradually decreases, the compression ratio gradually increases, and the entire region linearly changes.

(3) When the hot-pressing temperature is 135 °C and the pressure holding time is 180 s, the MEA is prepared with hot-pressing pressures of 0 MPa, 2.5 MPa, 5 MPa, 7.5 MPa, and 10 MPa, respectively, and its properties are studied. With the increase in the hot-pressing pressure, the peak power density of the titanium mesh-based MEA first increases and then gradually decreases. At room temperature, the peak power density appears when the hot-pressing pressure is 2.5 MPa; at 60 °C, the peak power density appears when the hot-pressing pressure is 5 MPa.

(4) When the hot-pressing pressure is 5 MPa and the pressure holding time is 180 s, the MEA is prepared at the hot-pressing temperatures of 115 °C, 135 °C, and 155 °C, respectively, and its properties are studied. With the increase in the hot-pressing temperature, the peak power density of the titanium mesh-based MEA first increases and then decreases, and the peak power density comes out when the hot-pressing temperature is 135 °C.

## Figures and Tables

**Table 1:** Experimental materials.

Name	Model/Quality Score	Manufacturer
Titanium mesh	0.5 × 1.2 diamond hole	Anping County Wire and Wire Mesh FACTORY, Henan, China
PtRu/XC-72R	40 wt.% Pt, 20 wt.% Ru	Johnson Matthey, London UK
Nafion membrane	117	Dupont, Wilmington, DE, USA
Pt/XC-72R	40 wt.% Pt	Johnson Matthey, London, UK
Nafion solution	5%	Dupont, Wilmington, DE, USA
XC-72	Vulcan XC-72	Cabot, Boston, MA, USA
PTFE solution	60%	Dupont, Wilmington, DE, USA
CH_3_CH_2_OH	≥99.7 wt.%	Shanghai Chemical Reagent Co., Ltd., Shanghai, China
H_2_SO_4_	≥98 wt.%	Shanghai Chemical Reagent Co., Ltd., Shanghai, China
H_2_O_2_	30 wt.%	Shanghai Chemical Reagent Co., Ltd., Shanghai, China
Deionized Water	18.25 MΩ·cm	Self-made

**Table 2:** The forming thickness and compressibility data of titanium mesh-based MEA under different hot-pressing pressures.

Sample	Hot-Pressing Pressure (MPa)	Thickness of MEA (μm)	Compression Ratio (%)
1	0	850	0.00
2	2.5	690	18.82
3	5	660	22.35
4	7.5	630	25.88
5	10	600	29.41

## Data Availability

Not applicable.
